# Particle beam therapy versus photon radiotherapy for extrahepatic biliary cancer—systemic review and meta-analysis

**DOI:** 10.1093/jrr/rrad015

**Published:** 2023-04-07

**Authors:** Hideya Yamazaki, Takuya Kimoto, Satoshi Teramukai, Kei Fujikawa, Kei Shibuya, Motohisa Suzuki, Kazuki Terashima, Takashi Iizumi, Masaru Wakatsuki, Osamu Suzuki, Takumi Fukumoto, Masayuki Ohtsuka

**Affiliations:** Department of Radiology, Graduate School of Medical Science, Kyoto Prefectural University of Medicine, 465 Kajiicho Kawaramachi Hirokoji, Kamigyo-ku, Kyoto 602-8566, Japan; Department of Radiology, Graduate School of Medical Science, Kyoto Prefectural University of Medicine, 465 Kajiicho Kawaramachi Hirokoji, Kamigyo-ku, Kyoto 602-8566, Japan; Department of Biostatistics, Graduate School of Medical Science, Kyoto Prefectural University of Medicine, 465 Kajiicho Kawaramachi Hirokoji, Kamigyo-ku, Kyoto 602-8566, Japan; Department of Biostatistics, Graduate School of Medical Science, Kyoto Prefectural University of Medicine, 465 Kajiicho Kawaramachi Hirokoji, Kamigyo-ku, Kyoto 602-8566, Japan; Gunma University Heavy Ion Medical Center, 3-39-15, Showa-machi, Maebashi, Gunma 371-8511, Japan; Department of Radiology, Southern Tohoku Proton Therapy Center, Koriyama City, Fukushima 963-8052, Japan; Department of Radiology, Hyogo Ion Beam Medical Center, 1-2-1 Kouto Shingu-cho, Tatsuno City 679-5165, Hyogo, Japan; Department of Radiation Oncology, Proton Medical Research Center, University of Tsukuba Hospital, Tsukuba, 305-8575, Japan; QST Hospital, National Institutes for Quantum and Radiological Science and Technology, Chiba, 263-0004, Japan; Osaka Heavy Ion Administration Company, Otemae, Chuo-ku, Osaka-city, Osaka, 540-0008, Japan; Department of Surgery, Division of Hepato-Biliary-Pancreatic Surgery, Kobe University Graduate School of Medicine, 7-5-2 Kusunoki-cho, Chuo-ku, Kobe city, Hyogo, 650-0017, Japan; Department of General Surgery, Chiba University Graduate School of Medicine, 1-8-1 Inohana, Chuo-ku, Chiba, 260-0856, Japan

**Keywords:** meta-analysis, systemic review, particle beam therapy, stereotactic body radiotherapy, intensity-modulated radiotherapy

## Abstract

Particle beam therapy (PT) is a potentially promising approach to the treatment of extrahepatic biliary cancer (EBC) because of its unique dose distribution using the Bragg peak. However, the superiority of PT to photon radiotherapy (XT) remains unclear. Therefore, we conducted a systematic review and meta-analysis to compare PT and XT for the treatment of EBC. The primary endpoint was overall survival (OS), which was pooled using a random-effects model. Nine articles comprising a total of 1558 patients (seven XT articles, *n* = 1488 patients; two PT articles, *n* = 70 patients) were screened. In addition, we compared the outcomes of XT and PT with the outcomes available from a prospective data registry (proton-net). The 1-year OS probability rates were 55, 65 and 72% for the XT group, PT group and PT registry, respectively. The 2-year OS probability rates were 26, 38 and 38% for the XT group, PT group and PT registry, respectively. The 3-year OS probability rates were 12, 35 and 18% for the XT group, PT group and PT registry, respectively. Although the difference between the 1-year OS rates of the XT group and PT registry was statistically significant, no other significant superiority was observed among these groups. In conclusion, the efficacy of PT was not superior to that of XT during this meta-analysis.

## INTRODUCTION

Biliary tract cancer is a rare disease. In Japan, 22 201 new patients were diagnosed with biliary tract cancer in 2018, and 17 773 died in 2020; furthermore, the reported 5-year relative survival probability rate was 24.5% in patients treated during 2009–11 [1]. Extrahepatic biliary cancer (EBC), which originates from the epithelial cells of the extrahepatic bile duct, consists of the gallbladder and cholangiocarcinoma of the perihilar or distal bile duct. Although surgery involving complete resection has curative potential, the prognosis of this disease is poor. This is because most patients have unresectable locally advanced or metastatic disease attributable to late presentation and nonspecific symptoms; in addition, the standard of care is the combined chemotherapy comprising gemcitabine and cisplatin, resulting in a median survival time (MST) of ~1 year [2]. For unresectable nonmetastatic EBC, radiation therapy (with or without chemotherapy) is the treatment of choice for controlling local disease progression, which is one of the main causes of treatment failure. Because of anxiety for toxicity of surrounding normal tissues, conventional radiotherapy cannot deliver high doses to the tumor, which is beneficial for tumor control.

Recently, several advanced technologies have been introduced in the field of radiotherapy, such as stereotactic body radiotherapy (SBRT) and intensity-modulated radiotherapy (IMRT). Particle beam therapy (PT) is a potentially promising approach to treating EBC because of its unique dose distribution using the Bragg peak. However, the superiority of PT to photon radiotherapy (XT) remains unclear. Therefore, to examine the usefulness of PT for biliary tract cancer, we performed a systematic review and meta-analysis to compare the outcomes associated with the use of PT and XT. Furthermore, this study aimed to determine whether PT is superior to XT for EBC.

## MATERIALS AND METHODS

Radiotherapy for EBC is indicated for unresectable cases without distant metastasis. It includes brachytherapy, intraoperative irradiation, 3D conformal radiation therapy (3D-CRT), SBRT and IMRT. During this study, 3D-CRT, which is most commonly used in Japan, and SBRT and IMRT, which have been increasingly used, were compared with PT as XT. In addition, the results of XT were compared with those available from the prospective data registry of all Proton Beam institutions (proton-net) [3].

EBC is a heterogeneous disease that differs by site (perihilar region, distal region, gallbladder) and background. Therefore, we analyzed perihilar region cancer as a specific subgroup because this site is observed in the largest number of patients with EBC treated with radiotherapy and is relatively more uniform than other regions.

**Fig. 1:**
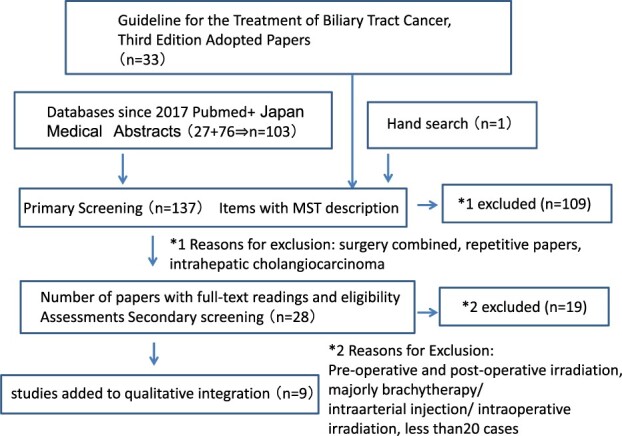
Literature search flowchart according to PRISMA statement.

The systematic review was performed according to the Preferred Reporting Items for Systematic Reviews and Meta-Analyses (PRISMA) guidelines [4]. The research methodology is illustrated in Fig. 1. We used a previous systematic review from the third edition of the biliary tract cancer practice guidelines (2019); the author of this study was a member of the guidelines committee and was responsible for the systematic review of radiotherapy [5]. The 33 studies selected from the practice guidelines were directly adopted for this analysis (Fig. 1). The details of the methodology are described in the guidelines using MeSH terms for the search items (Supplemental Table 1) [5]. Additional suitable articles were identified by screening two electronic databases (PubMed and Japan Medical Abstracts Library); this was performed after the period considered by the guidelines (May 2017–August 2020) by the librarian of Gunma University using the same MeSH terms [5]. In addition, one study was added after a manual search was performed. We excluded studies in which preoperative irradiation, postoperative irradiation, intraoperative irradiation, intra-arterial chemotherapy and brachytherapy were the main treatments. Furthermore, articles with a small number of patients (<20 patients) were excluded. Nine articles were selected after primary and secondary screenings of the 137 articles. Data regarding patients, diseases and treatment characteristics, as well as survival probability, local control probability and toxicity incidence were collected and reported using descriptive statistics. The primary endpoint was the overall survival (OS) probability.

### Statistical analysis

We conducted a random-effects meta-analysis [6] to pool the OS probabilities of PT and XT articles. ${\hat{S}}_k(t)$ was the estimate of the OS probabilities of the kth (k = 1, 2, …, 9) eligible articles at time t (t = 1, 2 and 3 years). When ${\hat{S}}_k(t)$ could not be obtained from the XT and PT articles, the values were imputed from the hazard estimated from the ${\hat{S}}_k(t)$ at the available time points under the assumption of an exponential distribution. The variance in ${\hat{S}}_k(t)$ was estimated as follows [7]: if the size of the risk set at time t (${n}_{kt}$) was available from the articles, then we used the values; however, if it was not available, then we estimated it as ${n}_{kt}={n}_k{\pi}_t{\hat{S}}_k(t)$. Here, ${n}_k$ was the total number of subjects and ${\pi}_t$ was a parameter that represented the proportion of noncensoring at time t. From the articles in which ${n}_{kt}$ was available, ${\pi}_t$ was obtained as ${\pi}_t=\frac{1}{k}\sum_k\frac{n_{kt}}{n_k{\hat{S}}_k(t)}$. Using the obtained ${n}_{kt}$, we estimated ${\eta}_k(t)$ as follows:


$$ {\eta}_k(t)=\sum_{{t_k}_{(i)}\le t}\frac{d_{ki}}{n_{ki}\left({n}_{ki}-{d}_{ki}\right)}\simeq{\pi}_t\sum_{j={n}_{kt}}^{n_k}\frac{1}{j\left(j-1\right)}, $$

where ${t_k}_{(i)}$ is the ordinal statistic, ${d}_{ki}$ is the number of events occurring at time ${t_k}_{(i)}$ and ${n}_{ki}$ is the size of the risk set immediately before time ${t_k}_{(i)}$. Then, the estimated ${\eta}_k(t)$ was used to estimate the variance of $\log \left(-\log \left({\hat{S}}_k(t)\right)\right)$ as follows:


$$ \widehat{Var}\left[\log \left(-\log \left({\hat{S}}_k(t)\right)\right)\right]={\eta}_k(t){\left(\log \left({\hat{S}}_k(t)\right)\right)}^{-2}. $$

Therefore, we obtained $\log \left(-\log \left({\hat{S}}_k(t)\right)\right)$ and $\widehat{Var}[\log(-\log ({\hat{S}}_k(t)))]$ for all k, performed a random-effects meta-analysis and estimated the pooled OS probabilities for XT (${\hat{S}}_X(t)$) and PT (${\hat{S}}_P(t)$) by performing (exp(−exp(·)) transformation. We estimated the difference between ${\hat{S}}_P(t)$ and ${\hat{S}}_X(t)$ and the 95% confidence interval (CI) as follows:


$$ \left({\hat{S}}_P(t)-{\hat{S}}_X(t)\right)\pm 1.96\sqrt{Var\left({\hat{S}}_P(t)\right)+ Var\left({\hat{S}}_X(t)\right)}. $$

The null hypothesis of ${S}_P(t)-{S}_X(t)=0$ was tested using a two-tailed significance level of 5%. In addition to the pooled estimate ${\hat{S}}_P(t)$, the OS probabilities of the PT group were compared when estimated from the registry data.

Statistical analyses were conducted using R software version 4.1.1 (http://R-project.org; The R Foundation for Statistical Computing, Vienna, Austria) and StatView 5.0 statistical software (SAS Institute, Cary, NC, USA). The R meta library was used to perform the meta-analysis (version 5.2.0). Statistical significance was set at *P* < 0.05.

## RESULTS

### Total population

We included nine articles (Fig. 1) (Table 1) in the systemic review (2 PT articles and 7 XT articles). One 3D-CRT article was a prospective phase II study, whereas the others were retrospective studies. We could not find an article that compared PT and XT.

The mean age of the population who received XT for EBC was 69.7 years (weighted by the number of patients), and the male-to-female ratio was 1:0.76, and Performance status 0–1 was 78.3%. Generally, when using 3D-CRT, the major schedule comprised 50 Gy in 2 Gy/fraction and additional dose escalation using brachytherapy or intraoperative irradiation. When using SBRT, a dose of 45 Gy in 3 Gy/fraction was administered. Chemotherapy is sometimes administered in neoadjuvant, concurrent or adjuvant settings. The MST ranged from 10 to 18.7 months, with 2-year survival probabilities of 15–30%. The 2-year local control probabilities ranged from 29 to 50%. The incidence of grade 3 or higher toxicity was 11–28%.

**Table 1:** Selected literatures by systemic review to compare the outcome between PT and XT

	Authors (ref no)	PY	Study	Treatment	Patient number	MST	2-year OS	2-year LC	Toxicity grade 3≤
XT	Yoshioka *et al*. [8]	2014	Retro_multi	3D-CRT^a^	286^d^	15	27%	NA	NA
	Tan *et al*. [9]	2015	Retro_single	3D-CRT	25	12	18%^c^	NA	NA
	Autorino *et al*. [10]	2016	Phase II	3D-CRT (wGEM)	27	14	27%	29%	Acute GI 18.5%
	Pollom *et al*. [11]	2017	SEER USA Population-based^e^	3D-CRT	451^d^	10^d^	15%^c^	NA	NA
	Torgeson *et al*. [12]	2017	NCDB USA Population-based^e^	3D-CRT	1070	14.5	36%^c^	NA	NA
	Elganainy *et al*. [13]	2018	Retro_single	3D-CRT^b^	80	18.7	33%^c^	50%^c^	Acute GI 11% Other 15% Late 28%
	Kopek *et al*. [14]	2010	Retro_single	SBRT	27^d^	10.6	16%^c^	NA	Ulcer 22%Stenosis11%
PT	Terashima *et al*. [15]	2018	Retro_single	Proton・Carbon	41	23	50%^c^	1y88%	NA
	Kasuya *et al*. [16]	2019	Retro_multi	Carbon	29	12.6	26.3%	58.2%	8.9%^d^
	Proton Beam Registry (Proton-net) [3]	2022	Pros-multi	Proton	93	20.1	38%	67%	15%

PY = publish year, GEM = gemcitabine, NA = not available, MST = median survival time, OS = overall survival rate, LC = local control rate

^a^Including intraoperative radiotherapy・Brachytherapy.

^b^Including 1 proton.

^c^Estimate from figure.

^d^Including intrahepatic bile duct cancer.

^e^Study on public database.

Regarding PT, 50–76 Gy was administered in 4–26 fractions, with an MST of 12.6–23 months and 2-year survival probabilities of 26.3–50%. The incidence of grade 3 or higher adverse events was 8.9%.

For comparison, the Proton Beam Registry was used to analyze 93 enrolled patients with unresectable EBC. We used three schedules (i) Gastrointestinal proximity type used 50–60 Gy (RBE)/25–30 fraction, (ii) Simultaneous integrated boost type used 67.5 Gy (RBE)/ 25 fraction, (iii) Perihilar type used 70.2–72.6 Gy (RBE)/22–26 fraction. The 2-year survival probability was 37.8%, and the MST was 20.1 months (range, 15.5–23.5 months). The 2-year local control probability was 66.5% (range, 48.4–79.5%). These results are summarized in Table 2.

**Table 2:** Summary of outcomes to compare the outcome between PT and XT

Treatment	MST (months)	2-year OS	2-year LC	Toxicity (G3≤)
XT (3DCRT–SBRT)	10–18.7	15–36%	29–50%	18.5–33%
PT	12.6–23	26.3–50%	58.2%	8.9%
PT Registry (proton)	20.1	38%	67%	15%

A meta-analysis of 1-year, 2-year and 3-year OS for EBC was performed, and the results were compared with the results of the PT group and PT registry (Table 3). The 1-year OS probabilities were 55, 65 and 72% for the XT group, PT group (XT group vs PT group: difference, 11%; 95% CI, −6 to 27%; *P* = 0.204) and PT registry (XT group vs PT registry: difference, 17%; 95% CI, 4–31%; *P* = 0.012), respectively (Supplemental Fig. 1). The 2-year OS probabilities were 26, 38 and 38% for the XT group, PT group (XT group vs PT group: difference, 13%; 95% CI, −12 to 38%; *P* = 0.313) and PT registry (XT group vs PT registry: difference, 12%; 95% CI, −1 to 26%; *P* = 0.069), respectively. The 3-year OS probabilities were 12, 35 and 18% for the XT group, PT group (XT group vs PT group: difference, 23%; 95% CI, −1 to 46%; *P* = 0.058) and PT registry (XT group vs PT registry: difference, 6%; 95% CI, −6 to 17%; *P* = 0.328), respectively.

**Table 3:** A meta-analysis of 1-, 2- and 3-year survival rates for BTC

Treatment	1-year OS	Difference; 95% CI	*P*-value	2-year OS	Difference, 95% CI	*P*-value	3-year OS	Difference, 95% CI	*P*-value
XT (3DCRT–SBRT) (7 articles)	55%			26%			12%		
PT (2 articles)	65%	^*^11%; −6% to 27%	^*^ *P* = 0.204	38%	^*^13%; −12% to 38%	^*^ *P* = 0.313	35%	^*^23%; −1% to 46%	^*^ *P* = 0.058
PT Registry (proton)	72%	^**^17%; 4–31%	^**^ *P* = 0.012	38%	^**^12%; −1% to 26%;	^**^ *P* = 0.069	18%	^**^6%; −6% to 17%	^**^ *P* = 0.328

CI = confidence interval.

^*^XT vs PT.

^**^XT vs PT registry.

Although a statistically significant difference in the 1-year OS probabilities of the XT group and PT registry (*P* = 0.012) was observed, no other statistically significant difference was observed between the groups. The incidence rates of grade 3 or higher toxicity were 18.5–33% in the XT group and 2.9–15% in the PT group and PT registry. PT for EBC showed a favorable trend for OS compared with XT, but no clear superiority was observed. There was also a trend toward a lower incidence of grade 3 or higher toxicity when PT was used.

### Perihilar region

Articles in which the perihilar region comprised more than two-third of cases were selected (Table 4). The mean age of the population who received XT for unresectable hilar region cholangiocarcinoma was 66.9 years (mean and median weighted by the number of cases), and the male-to-female ratio was 1:0.63, Performance status 0–1 was 86.9%. The MST was 10.6–18.7 months, and the 2-year OS ranged from 15 to 30%. The local control probability ranged from 29 to 50%. The rate of grade 3 or higher adverse events ranged from 11 to 28%.

**Table 4:** Selected literatures by systemic review to compare the outcome between PT and XT for perihilar cancer

	Authors (ref no)	PY	Study	Treatment	Patient number (Perihilar %)	MST	2-year OS	2-year LC	Toxicity grade 3≤
XT	Tan *et al*. [9]	2015	Retro_single	3D-CRT	25 (100%)	12	18%^b^	NA	NA
	Autorino *et al*. [10]	2016	Phase II	3D-CRT (wGEM)	18 (67%)	14	27%	29%	Acute GI 18.5%
	Elganainy *et al*. [13]	2018	Retro_single	3D-CRT^a^	62 (78%)	18.7	33%^b^	50%^b^	Acute GI 11% Other 15% Late 28%
	Kopek *et al*. [14]	2010	Retro_single	SBRT	26 (96%)	10.6	16%^b^	NA	Ulcer 22% Stenosis 11%
PT	Terashima *et al*. [15]	2018	Retro_single	Proton・Carbon	41 (100%)	23	50%^b^	1y88%	NA
	Kasuya *et al*. [16]	2019	Retro_multi	Carbon	29 (100%)	12.6	26.3%	58.2%	8.9%^c^
	Proton Beam Registry (Proton-net) [3]	2022	Pros-multi	Proton	55 (59%)	20.1	39%	68%	20%

wGEM = with gemcitabine.

^a^Including 1 proton.

^b^Estimate from figure.

^c^Including intrahepatic.

Regarding PT, the prescribed dose of 50–76 Gy was administered over the course of 4–26 sessions. The MST was 12.6–23 months, and the 2-year survival probability ranged from 26.3 to 50%. The incidence of grade 3 or higher toxicity was 8.9% (Table 5).

**Table 5:** Summary of outcomes to compare the outcome between PT and XT in perihilar region

Treatment	MST (months)	2-year OS	2-year LC	Toxicity (G3≤)
XT (3DCRT–SBRT)	10.6–18.7	16–33%	29–50%	18.5–33%
PT	12.6–23	26.3–50%	58.2%	8.9%
PT Registry (proton)	20.1	39%	68%	20%

For further comparison, 55 cases of unresectable hilar cholangiocarcinoma were analyzed using the PT Registry (Proton Beam Registry). The MST was 20.1 months, and the 2-year survival probability was 38.9%. The 2-year local control probability was 68.5% (range, 42.1–84.8%). The incidence of grade 3 or higher toxicity was 20%.

A meta-analysis of 1-year, 2-year and 3-year survival probabilities for unresectable hilar region cholangiocarcinoma was performed, and those results were compared with the results from a particle beam registry (proton-net) (Table 6).

**Table 6:** A meta-analysis of 1-, 2- and 3-year survival rates for perihilar region cancer

Treatment	1-year OS	Difference; 95% CI	*P*-value	2-year OS	Difference; 95% CI	*P* value	3-year OS	Difference; 95% CI	*P* value
XT (3DCRT–SBRT) (4 articles)	56%			27%			11%		
PT (2 articles)	65%	^*^9%; −9% to 28%	^*^ *P* = 0.331	38%	^*^12%; −14% to 37%	^*^ *P* = 0.376	35%	^*^24%; 0.3% to 47%	^*^ *P* = 0.047
PT Registry (proton)	69%	^**^13%; −5% to 31%	^**^ *P* = 0.17	39%	^**^12%; −5% to 29%	^**^ *P* = 0.167	14%	^**^3%; −10% to 16%	^**^ *P* = 0.641

^*^XT vs PT.

^**^XT vs PT registry.

The 1-year OS probabilities were 56, 65 and 69% for the XT group, PT group (XT group vs PT group: difference, 9%; 95% CI, −9 to 28%; *P* = 0.331) and PT registry (XT group vs PT registry: difference, 13%; 95% CI, −5 to 31%; *P* = 0.170), respectively. The 2-year OS probabilities were 27, 38 and 39% for the XT group, PT group (XT group vs PT group: difference, 12%; 95% CI, −14 to 37%; *P* = 0.376) and PT registry (XT group vs PT registry: difference, 12%; 95% CI, −5 to 29%; *P* = 0.167), respectively. The 3-year OS probabilities were 11, 35 and 14% for the XT group, PT group (XT group vs PT group: difference, 24%; 95% CI, 0.3–47%; *P* = 0.047) and PT registry (XT group vs PT registry: the difference was 3%; 95% CI, −10 to 16%; *P* = 0.641), respectively. No statistically significant difference in the OS probabilities was observed among the groups except between the XT group vs. PT group in 3-year OS.

## DISCUSSION

This analysis compared the efficacy of PT and XT when using a superior PT dose distribution for EBC. An escalated dose or radiation can improve the prolonged local control probability, resulting in better survival. To our knowledge, this is the first study to compare the outcomes of PT and XT for EBC, and we found equivalent outcomes in terms of OS using PT and XT.

Based on the ABC-02 trial, the standard of care for unresectable EBC has been the systemic therapy using gemcitabine and cisplatin, which resulted in an MST of 8–12 months for 76% of disease cases with distant metastasis. Several studies of radiotherapy have observed its superiority over best supportive care [17, 18]; however, there is little evidence indicating that radiotherapy (with or without chemotherapy) is more beneficial than chemotherapy alone. The clinical question of the guidelines for the treatment of biliary tract cancer (third edition) regarding whether radiotherapy or chemoradiotherapy should be administered for unresectable biliary tract cancer stated that the evidence of the benefits of radiotherapy and chemoradiotherapy is insufficient at present, and that no clear recommendation can be made [5].

Escalating the radiation dose has the potential to improve outcomes [19–21]. Recent advanced technologies, such as SBRT and IMRT, and PT could be administered with an increased target dose without unnecessary irradiation to the surrounding normal tissue [13]. IMRT is a good alternative to 3D-CRT because it can reduce normal tissue toxicity and improve the dose distribution [13, 22]. Furthermore, SBRT could deliver a higher prescribed dose per fraction with precise accuracy and a short treatment period [14, 21]. These techniques have been explored as potentially curative radiotherapy strategies for patients with EBC [14, 21]. Lee *et al*. performed a systemic review of SBRT and reported an MST of 13 months and a late toxicity rate of 0–20% [23]. The use of a particle beam, which has a physical advantage over standard XT using the Bragg peak, is associated with an MST of 12–23 months for EBC [15, 24, 25]. These advantages are more pronounced in patients with intrahepatic cholangiocarcinoma [26]. The 3-year OS probability for patients receiving a biological equivalent dose (BED) >80.5 Gy was 73%; however, it was only 38% for those receiving lower doses. Furthermore, the 3-year local control probability was significantly higher (78%) with a BED >80.5 Gy than with lower doses [26]. Unlike intrahepatic biliary cancer, EBC is located close to the bowel; therefore, the prescribed dose to the tumor should be limited to avoid higher doses in the gastrointestinal tract. Consequently, according to the United States Astromodel Policy, biliary tract cancer is considered Group 2 (more evidence is needed, and PT is recommended when it is difficult to treat with the XT dose distribution) [27].

This study had some limitations. We could not find any prospective randomized trials comparing the outcomes of PT and XT. The few comparable studies that exist evaluated a limited number of patients and were mostly retrospective. Moreover, selection bias in the pretreatment decisions was possible not only for XT but also for PT. Finally, the 2-year and 3-year survival probabilities may not be appropriate endpoints for the comparison of the survival probabilities of PT and XT for EBC because the MST was within 10–21 months. In addition, the 1-year survival probability of the PT registry was significantly improved compared with that of the XT group. At last, the part of statistical method used in this paper [7] was not established in statistical journal but proposed recently to compare the treatment outcome and used in several studies. Despite these limitations, this is the first comprehensive analysis to examine the role of PT compared with that of XT. Further exploration of the role of PT as part of a multimodal curative treatment strategy is required.

## CONCLUSION

The efficacy of PT was not superior to that of XT during this meta-analysis.

## DATA AVAILABILITY

The data of this study can be obtained from the author upon reasonable request.

## CONFLICT OF INTEREST

None declared.

## FUNDING

This work was supported by Hokkaido University (Functional enhancement promotion expenses by the Ministry of Education, Culture, Sports, Science and Technology) and AMED under Grant Number JP16lm0103004.

## Supplementary Material

Supplemental_Figure_rrad015

Supplemental_Table_1_rrad015
