# Supplementary material for: Particle beam therapy versus photon radiotherapy for extrahepatic biliary cancer—systemic review and meta-analysis
Source: J Radiat Res. 2023 Apr 7;64(Suppl 1):i34–40. doi: 10.1093/jrr/rrad015 (PMC10278884; doi:10.1093/jrr/rrad015)
Supplement: Supplemental_Figure_rrad015 [file supplemental_figure_rrad015.docx]

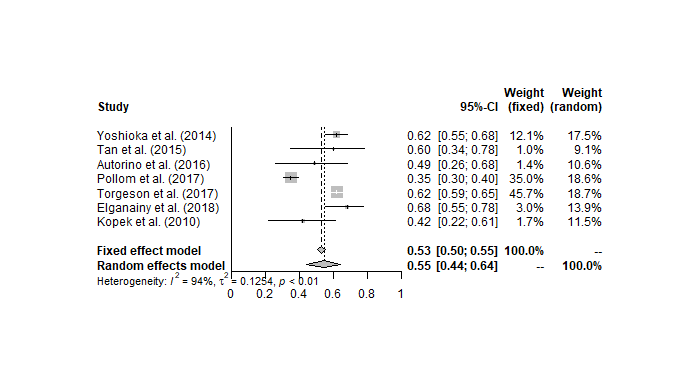


Supplemental Figure 1. Forest plot of 1-year OS for XT (3DCRT～SBRT) in total population


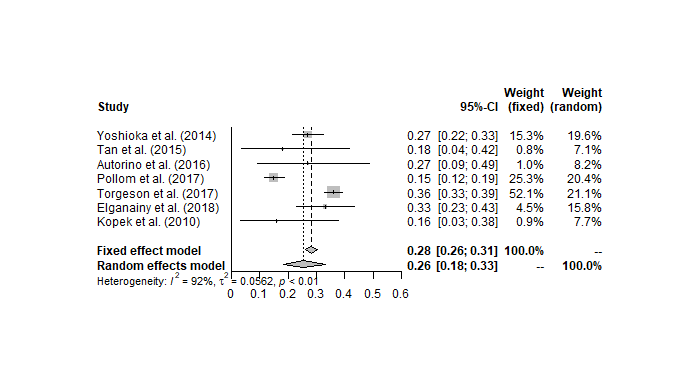


Supplemental Figure 2. Forest plot of 2-year OS for XT (3DCRT～SBRT) in total population


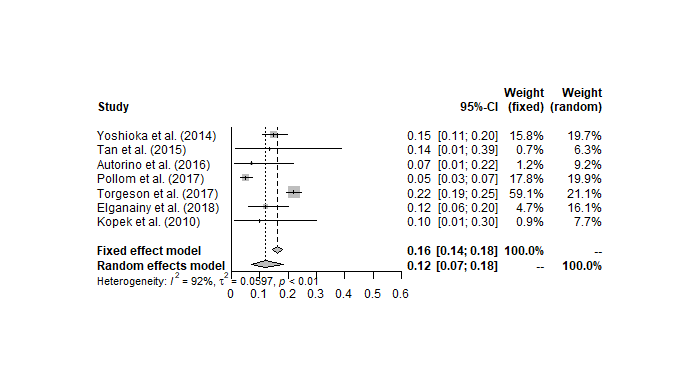


Supplemental Figure 3. Forest plot of 3-year OS for XT (3DCRT～SBRT) in total population


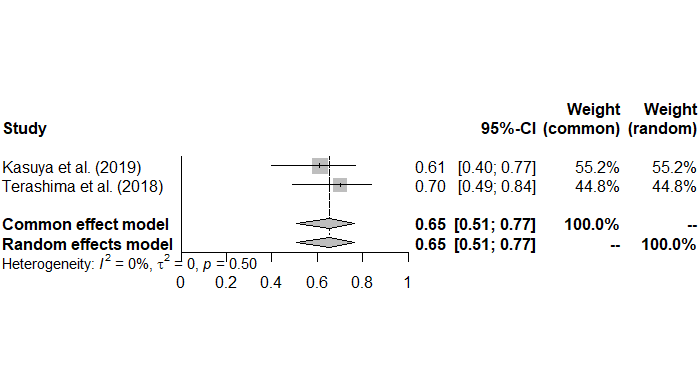


Supplemental Figure 4. Forest plot of 1-year OS for PT in total population (same in perihilar region)


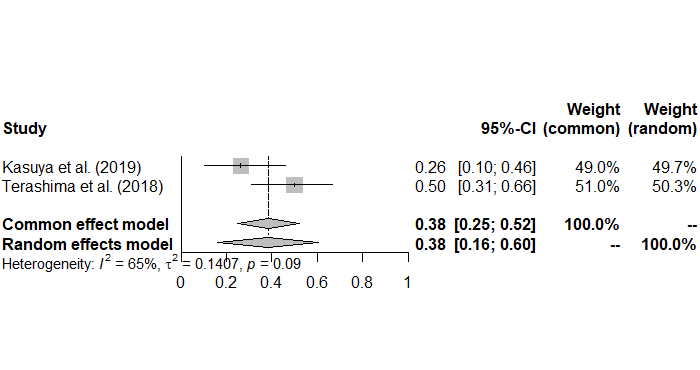


Supplemental Figure 5. Forest plots of 2-year OS for PT in total population (same in perihilar region)


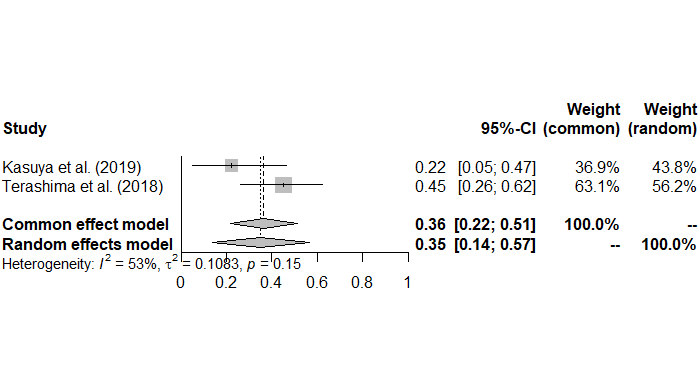
Supplemental Figure 6. Forest plots of 3-year OS for PT in total population (same in perihilar region)


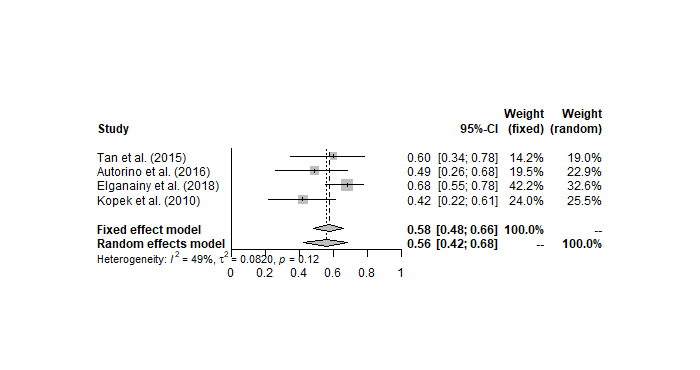


Supplemental Figure 7. Forest plot of 1-year OS for XT (3DCRT～SBRT) in perihilar region


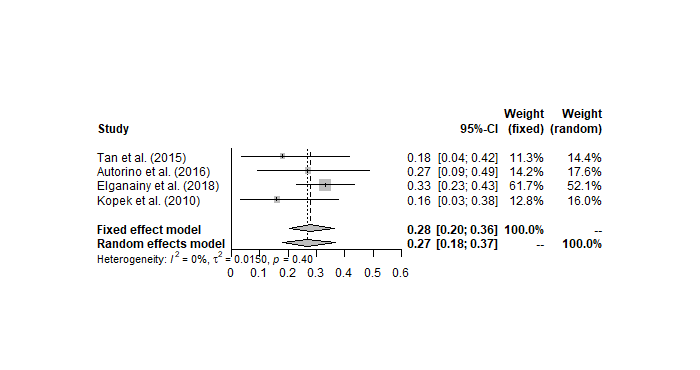
Supplemental Figure 8. Forest plot of 2-year OS for XT (3DCRT～SBRT) in perihilar region


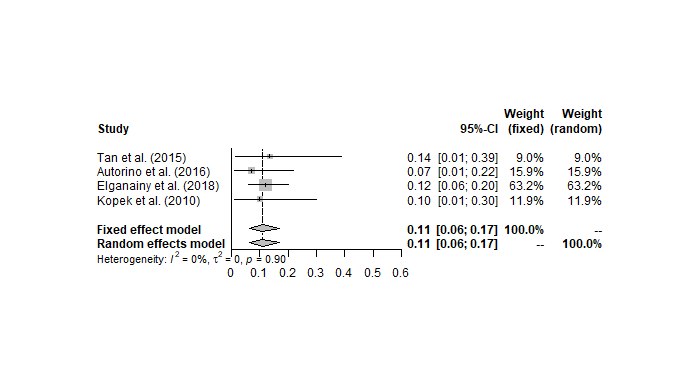


Supplemental Figure 9. Forest plot of 3-year OS for XT (3DCRT～SBRT) in perihilar region
